# Lipid phosphatase SHIP2 functions as oncogene in colorectal cancer by regulating PKB activation

**DOI:** 10.18632/oncotarget.12321

**Published:** 2016-09-28

**Authors:** Elmer Hoekstra, Asha M. Das, Marcella Willemsen, Marloes Swets, Peter J.K. Kuppen, Christien J. van der Woude, Marco J. Bruno, Jigisha P. Shah, Timo L.M. ten Hagen, John D. Chisholm, William G. Kerr, Maikel P. Peppelenbosch, Gwenny M. Fuhler

**Affiliations:** ^1^ Department of Gastroenterology and Hepatology, Erasmus MC, University Medical Center Rotterdam, Rotterdam, The Netherlands; ^2^ Department of Surgery, Section Surgical Oncology, Laboratory Experimental Surgical Oncology, Erasmus MC, University Medical Center Rotterdam, Rotterdam, The Netherlands; ^3^ Department of Surgery, Leiden University Medical Center, Leiden, The Netherlands; ^4^ Department of Chemistry, Syracuse University, Syracuse, New York, United States of America; ^5^ Department of Microbiology and Immunology, State University of New York (SUNY) Upstate Medical University, Syracuse, New York, United States of America

**Keywords:** colorectal cancer, SHIP2, phosphatases, migration, small molecule inhibitor

## Abstract

Colorectal cancer (CRC) is the second most common cause of cancer-related death, encouraging the search for novel therapeutic targets affecting tumor cell proliferation and migration. These cellular processes are under tight control of two opposing groups of enzymes; kinases and phosphatases. Aberrant activity of kinases is observed in many forms of cancer and as phosphatases counteract such “oncogenic” kinases, it is generally assumed that phosphatases function as tumor suppressors. However, emerging evidence suggests that the lipid phosphatase SH2-domain-containing 5 inositol phosphatase (SHIP2), encoded by the *INPPL1* gene, may act as an oncogene. Just like the well-known tumor suppressor gene Phosphatase and Tensin Homolog (PTEN) it hydrolyses phosphatidylinositol (3,4,5) triphosphate (PI(3,4,5)P3). However, unlike PTEN, the reaction product is PI(3,4)P2, which is required for full activation of the downstream protein kinase B (PKB/Akt), suggesting that SHIP2, in contrast to PTEN, could have a tumor initiating role through PKB activation. In this work, we investigated the role of SHIP2 in colorectal cancer. We found that SHIP2 and *INPPL1* expression is increased in colorectal cancer tissue in comparison to adjacent normal tissue, and this is correlated with decreased patient survival. Moreover, SHIP2 is more active in colorectal cancer tissue, suggesting that SHIP2 can induce oncogenesis in colonic epithelial cells. Furthermore, *in vitro* experiments performed on colorectal cancer cell lines shows an oncogenic role for SHIP2, by enhancing chemoresistance, cell migration, and cell invasion. Together, these data indicate that SHIP2 expression contributes to the malignant potential of colorectal cancer, providing a possible target in the fight against this devastating disease.

## INTRODUCTION

Colorectal cancer (CRC) is the second most common malignancy in women and the third most common malignancy in men worldwide [1]. Approximately 10% of all newly diagnosed malignancies are colorectal cancers, equivalent to 1.2 million cases each year. Although CRC mortality has been declining in the last decades, due to improvements in screening techniques and CRC treatment, it is still the second most common cause of cancer-related death. This is mainly due to the fact that around 20% of colorectal cancer patients are diagnosed with metastatic disease, drastically dropping their 5-year survival from 90% in non-metastatic patients, to a mere 12% [1]. Originally, the only medical intervention available consisted of chemotherapy, commonly antimetabolite drugs interfering in biosynthetic processes. These compounds destroy rapidly dividing cells, often by interfering in purine and pyrimidine synthesis; however, they do not specifically target cancer cells alone, resulting in a number of side-effects. In the last decades, more specific ‘targeted therapies’ have made their appearance in the clinic. These inhibitors mostly affect cell proliferation by interfering in cell cycle progression by targeting signal transduction pathways affected in CRC. In particular, inhibitors of the Epidermal Growth Factor Receptor (EGFR), which is often over-expressed in CRC, have shown promise in this disease [2–4]. EGFR is a receptor with enzymatic kinase activity, whose activity results in phosphorylation and activation of downstream signaling cascades such as the Ras-Raf-MEK-ERK and PI3K-PKB/Akt pathways [5]. However, 4 out of 10 CRCs harbor an activating mutation in the Ras gene [6], rendering the use of upstream EGFR inhibitors ineffective [7]. Pan-kinase inhibitors (e.g. Regorafenib [8]) or selective kinase inhibitors (e.g. PI3K/AKT inhibitors [9]) are therefore currently being tested. As phosphorylation of proteins and lipids is essential for virtually all cellular functions, tight control is required and provided by the counteracting enzyme class of phosphatases. While kinases have so far been targeted for treatment, phosphatases are generally regarded as tumor suppressors and have for the most part been disregarded in cancer research. However, there is emerging evidence that phosphatases can also act as activators of signaling, thereby presenting them as possible oncogenes and targets for treatment [10–13].

The SH2-domain-containing 5 inositol phosphatase (SHIP2), encoded by the *INPPL1* gene, is a lipid phosphatase which acts in PI3K-PKB-mTOR pathway [14]. PI3K phosphorylates membrane bound inositol lipids to produce phosphatidylinositol(3,4,5) triphosphate (PIP_3_), which subsequently recruits and allows activation of PKB [9,15]. Hydrolysis of PIP_3_ is generally assumed to terminate PKB signaling, as is the case for the well-known tumor suppressor gene Phosphatase and Tensin Homolog (PTEN) [16] However, where PTEN produces PI(4,5)P_2_, hydrolysis of PIP_3_ by SHIP2 produces PIP(3,4)P_2_, which was recently shown to have a high affinity for PKB and to be required for full PKB activation [17, 18]. Thus, both PIP_3_ and PI(3,4)P_2_ are suggested to play a role in cancer development [12, 13, 19], identifying SHIP2 as a possible oncogene rather than tumor suppressor. The importance of PI(3,4)P_2_ as activator of oncogenic signaling is further supported by the fact that hydrolysis of PI(3,4)P_2_ into PI(3)P by INPP4B reduces tumorigenesis [20, 21].

This study investigates the role of the SHIP2 phosphatase in colorectal cancer. We show that SHIP2 and *INPPL1* expression are increased in colorectal cancer tissue in comparison to adjacent normal tissue, which results in worse patient outcome. Importantly, SHIP2 enzymatic activity is also enhanced in colorectal cancer tissue. Furthermore, *in vitro* experiments performed on colorectal cancer cell lines show an oncogenic role for SHIP2, through enhancement of chemoresistance, cell migration, and cell invasion. Together, these studies suggest that SHIP2 expression contributes to the malignant potential of colorectal cancer, providing a possible target in the fight against this devastating disease.

## RESULTS

### INPPL1 mRNA expression is increased in colorectal adenomas and carcinomas

**Figure 1:**
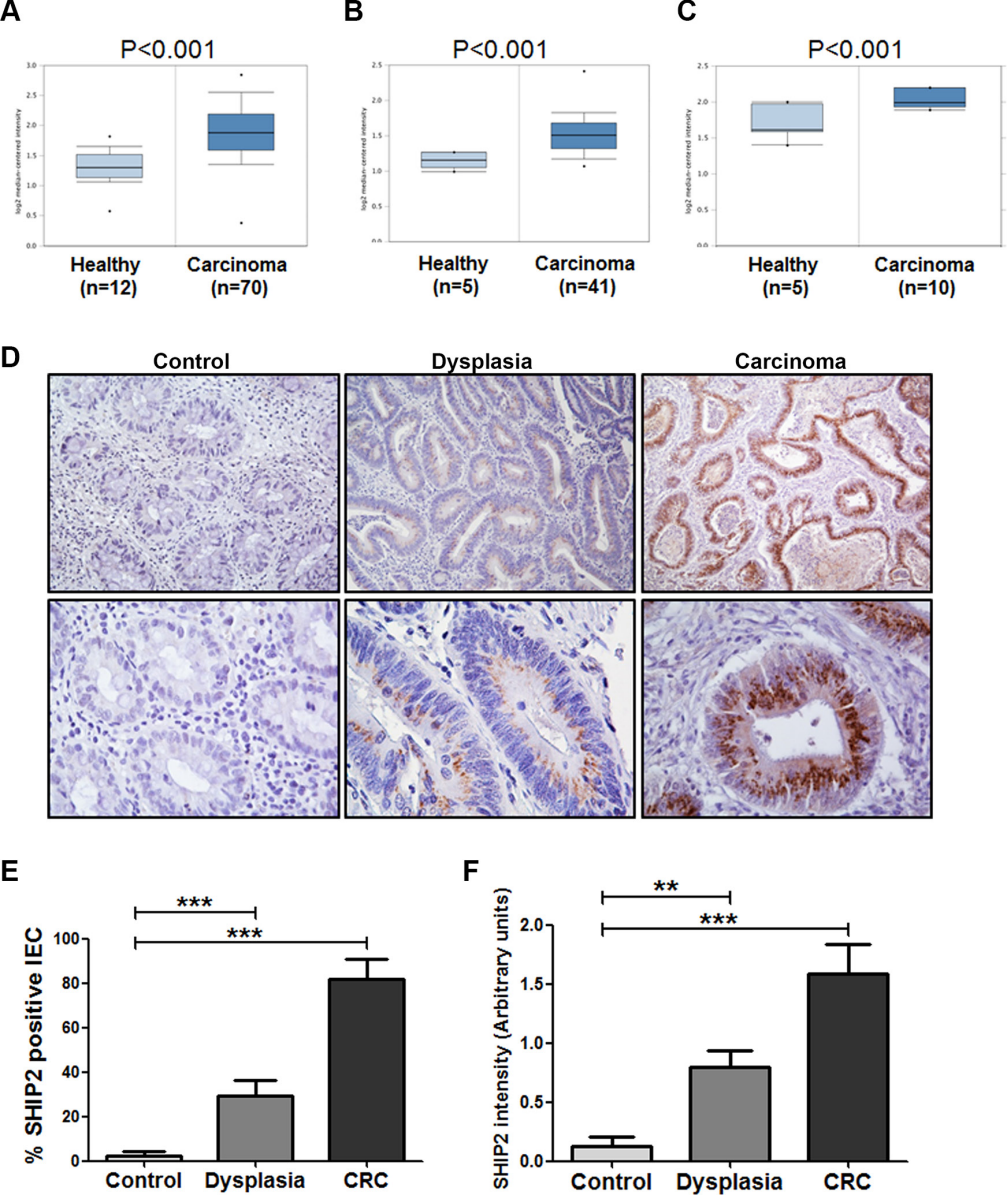
INPPL1 mRNA and SHIP2 protein expression are increased in colorectal dysplasia and carcinoma as compared to non-dysplastic tissue (**A**–**C**) Using publicly available gene expression data from Oncomine, *INPPL1* expression was analyzed in carcinoma tissue compared to adjacent normal colon tissue. Shown are the significant upregulation of INPPL1 in CRC in datasets from Hong *et al.* (A), Kaiser *et al.* (B) and Skrzypczakand *et al.* (C). (**D**) Tissues of patients dysplasia (*n* = 14), colorectal cancer (CRC, *n* = 11), and controls (inactive ulcerative colitis, *n* = 8), were stained for SHIP2 by immunohistochemistry. Representative examples (10× and 40× magnification) are shown. (**E**, **F**) SHIP2 staining was scored for percentage of positive intestinal epithelial cells as well as intensity of staining. (**p > 0.01; ***p > 0.001).

To understand the role of SHIP2 in colorectal cancer, we first investigated the expression levels of the SHIP2 encoding gene *INPPL1* using publicly available microarray datasets from the Oncomine Cancer Microarray database (http://www.oncomine.org/). In 3 out of 10 datasets, a significantly increased *INPPL1* expression in CRC was reported. In the study performed by Hong and colleagues [24], gene expression was compared between colorectal carcinomas (*n* = 70) and adjacent colonic tissues (*n* = 12). *INPPL1* mRNA expression was significantly increased in the carcinoma group (*P* < 0.001). Moreover, the SHIP2 encoding gene was again significantly increased in colorectal carcinoma compared to healthy colon in the studies by Kaiser et al. and Skrzypczakand and co-workers (*n* = 41 versus *n* = 5, and n=10 versus *n* = 5 respectively) [25, 26] (Figure 1A–1C). The remaining 7 data-sets did not reach statistical significance individually. However, in an overall comparison of all the available datasets, *INPPL1* expression was significantly increased (*p* = 0.041).

### SHIP2 protein is overexpressed in primary colorectal cancer samples

Next, we examined whether the increased *INPPL1* mRNA levels corresponded to increased SHIP2 protein levels. Therefore, immunohistochemistry was performed on microsections of biopsies of 14 dysplasia patients (9 low grade, 5 high grade), 11 colorectal cancer (CRC) patients, and 8 controls (inactive ulcerative colitis) (Figure 1D). SHIP2 expression in intestinal epithelial cells (IEC) was very limited (mean 3% ± 5) in cells of non-cancerous tissues. In contrast, SHIP2 expression was significantly increased in dysplastic tissues (29% ± 26 positive IEC), whereas up to 82% of SHIP2-positive IEC were found in CRC (*p* < 0.001) (Figure 1E). Likewise, SHIP2 staining intensity follows the same trend with intensity increasing with progressing levels of dysplasia (0.13 ± 0.23, 0.80 ± 0.53, 1.59 ± 0.80 in non-cancerous tissue, dysplasia and CRC respectively, *p* < 0.01) (Figure 1F).

**Figure 2:**
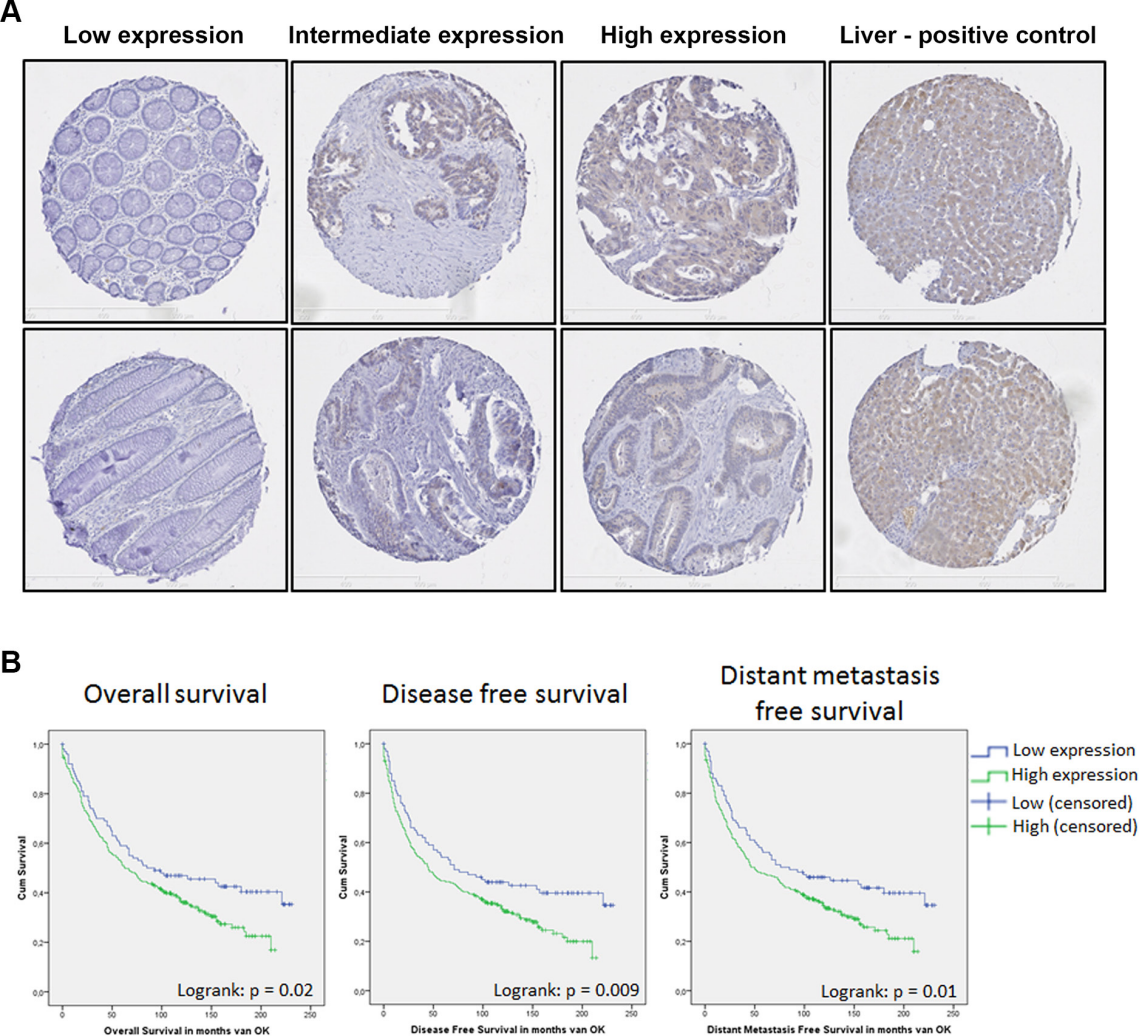
SHIP2 expression in a large cohort of CRC patients is correlated to a worse patient survival IHC analysis of SHIP2 on a tissue micro array (TMA) of colorectal cancer patients (*n* = 347) and healthy adjacent tissue (*n* = 206). (**A**) Representative stainings of tissue cores are shown, with liver tissue as positive control. (**B**) Using the previously published web-application “cut-off finder” we divided the patients in two groups based on percentage SHIP2-positive intestinal epithelial cells (High/Low). Kaplan meier curves for overall survival, disease free survival, and distant metastasis free survival reveal a significant correlation between high SHIP2 expression and these clinical parameters.

To further confirm these findings in a larger cohort, SHIP2 expression was analyzed using a previously described tissue micro array (TMA) containing 455 colorectal cancer patients, of which 347 patient samples of colorectal cancer and 246 (matched) healthy tissues could be analyzed (Figure 2A). We observed a significant increase of SHIP2 positive IECs in cancerous compared to non-cancerous tissue (*p* < 0.0001). For 206 patients we could analyze both the cancerous and the normal adjacent tissue of the same patient. Using paired testing, we found a significant increase in tumor tissue (57% versus 28%; *p* < 0.001 respectively), suggesting that increased SHIP2 expression in intestinal epithelial cells may contribute to oncogenic transformation.

Next, we investigated whether increased SHIP2 expression in CRC correlates to clinical characteristics, and whether this is predictive of poor patient outcome. First, we defined the correct cut-off value of SHIP2 expression, using the previously published web-application “cut-off finder” [27]. Based on ROC-curve analysis, the cut-off was defined at 38% positive intestinal epithelial cells on the TMA tissue cores, resulting in two groups (< 38%, defined as “low” and > 38%, defined as “high”), with 101 patients in the “low” group, and 246 patients in the “high” group. The clinicopathological characteristics of the patient cohort and their relation to SHIP2 expression levels are listed in Table 1. SHIP2 expression did not correlate to TNM-stage, Dukes' stage, tumor differentiation, tumor location, and PI3K-mutation status, but did correlate significantly to p53 protein expression and MSI-status (described previously in [22]).

**Table 1:** Patient characteristics of tissue micro array stained for SHIP2

Parameter	SHIP2 Low	SHIP2 high	Overall	*P*-value
**Number of patients**	101	246	347	
**Gender (M)**	50 (50%)	122 (50%)	172 (50%)	
**Age**	64.99	66.04	65.30	
**Location**				
Left-sided	58 (62%)	168 (72%)	226 (70%)	0.112
Right-sided	35 (38%)	66 (28%)	101 (30%)	
**TNM stadium (AJCC 5)**				
Stage I	25 (25%)	40 (17%)	65 (19%)	0.294
Stage II	36 (36%)	84 (35%)	120 (36%)	
Stage III	24 (24%)	69 (29%)	93 (28%)	
Stage IV	15 (15%)	45 (19%^)	60 (18%)	
**Differentiation tumor**				
Well	20 (26%)	37 (19%)	57 (21%)	0.454
Moderate	48 (62%)	134 (69%)	182 (67%)	
Poor	9 (12%)	37 (11%)	31 (11%)	
**Dukes stadium**				
A/B	62 (62%)	126 (53%)	188 (55%)	0.290
C	23 (23%)	68 (28%)	91 (27%)	
D	15 (15%)	45 (19%)	60 (18%)	
**MSI-status**				
MSS	58 (60%)	174 (75%)	232 (71%)	0.032
MSI-H	13 (14%)	23 (10%)	36 (11%)	
unknown	25 (26%)	36 (15%)	61 (19%)	
**p53**				
0–25%	59 (60%)	116 (48%)	175 (51%)	0.043
> 25%	39 (40%)	126 (52%)	165 (39%)	
**PIK3CA**				
WT	66 (65%)	195 (79%)	261 (75%)	0.683
Mutation	10 (10%)	25 (10%)	35 (10%)	
Missing	25 (25%)	26 (11%)	51 (15%)	

Kaplan Meier survival analysis revealed that high expression of SHIP2 is significantly inversely correlated to overall survival (OS; logrank *p* = 0.021), disease free survival (DFS; logrank *p* = 0.009), local recurrence free survival (LRFS; logrank *p* = 0.025), and distant metastasis free survival (DMFS; logrank = 0.01) (Figure 2B). Furthermore, OS and DFS were analyzed in a multivariable model, adjusting for the variables sex, age at time of operation, TNM stage, tumor location, and SHIP2 status. Significant variables in the univariate analysis were used in the final multivariable model. Significant independent predictors for both survival outcomes are age at time of operation and TNM-status. While high SHIP2 expression was not a significant independent predictor for OS, it was borderline significant for DFS (*p* = 0.059) (Tables 2–3).

**Table 2:** Uni- and multivariate analysis for overall survival

	Univariate		Multivariate	
	HR	*p*-value	HR	*p*-value
**SHIP2**	1.421(1.0–1.922)	**0.023**	1.232 (0.906–1.676)	0.183
**Gender (M)**	1.304 (1.044–1.629)	**0.019**		
**Age**	1.039 (1.028–1.05)	**< 0.001**	1.049 (1.035–1.063)	**< 0.001**
**TNM stage**				
stage 1		**< 0.001**		**< 0.001**
stage 2	1.386 (0.983–1.954)		1.635 (1.069–2.500)	
stage3	2.078 (1.459–2.959)		2.279 (1.474–3.523)	
stage 4	6.142 (4.199–8.985)		6.691 (4.246–10.546)	
**Dukes' stage**				
A/B		**< 0.001**		
C	1.696 (1.3-2.212)			
D	4.987 (3.698–6.726)			
**Differentation**	0.96 (0.825–1.117)	0.596		
**Adjuvant therapy**	1.056 (0.957–1.166)	0.279		
**Tumor location**	1.258 (0.988–1.601)	0.063		

**Table 3:** Uni- and multivariate analysis for disease free survival

	Univariate		Multivariate	
	HR	*p*-value	HR	*p*-value
**SHIP2**	1.474 (1.096–1.983)	**0.01**	1.337 (0.989–1.806)	**0.059**
**Gender (M)**	1.289 (1.037–1.604)	**0.022**		
**Age**	1.032 (1.021–1.042)	**< 0.001**	1.039 (1.026–1.051)	**< 0.001**
**TNM stage**				
stage 1		**< 0.001**		**< 0.001**
stage 2	1.398 (0.999–1.956)		1.544 (1.023–2.330)	
stage3	2.085 (1.474–2.949)		2.132 (1.398–3.251)	
stage 4	6.171 (4.232–8.999)		6.300 (4.004–9.816)	
**Dukes' stage**				
A/B		**< 0.001**		
C	1.69 (1.302–2.193)			
D	4.979 (3.694–6.71)			
**Differentation**	0.986 (0.852–1.141)	0.853		
**Adjuvant therapy**	1.036 (0.937–1.145)	0.494		
**Tumor location**	1.107 (0.872–1.405)	0.404		

### Intrinsic SHIP2 activity is increased in colorectal cancer samples

**Figure 3:**
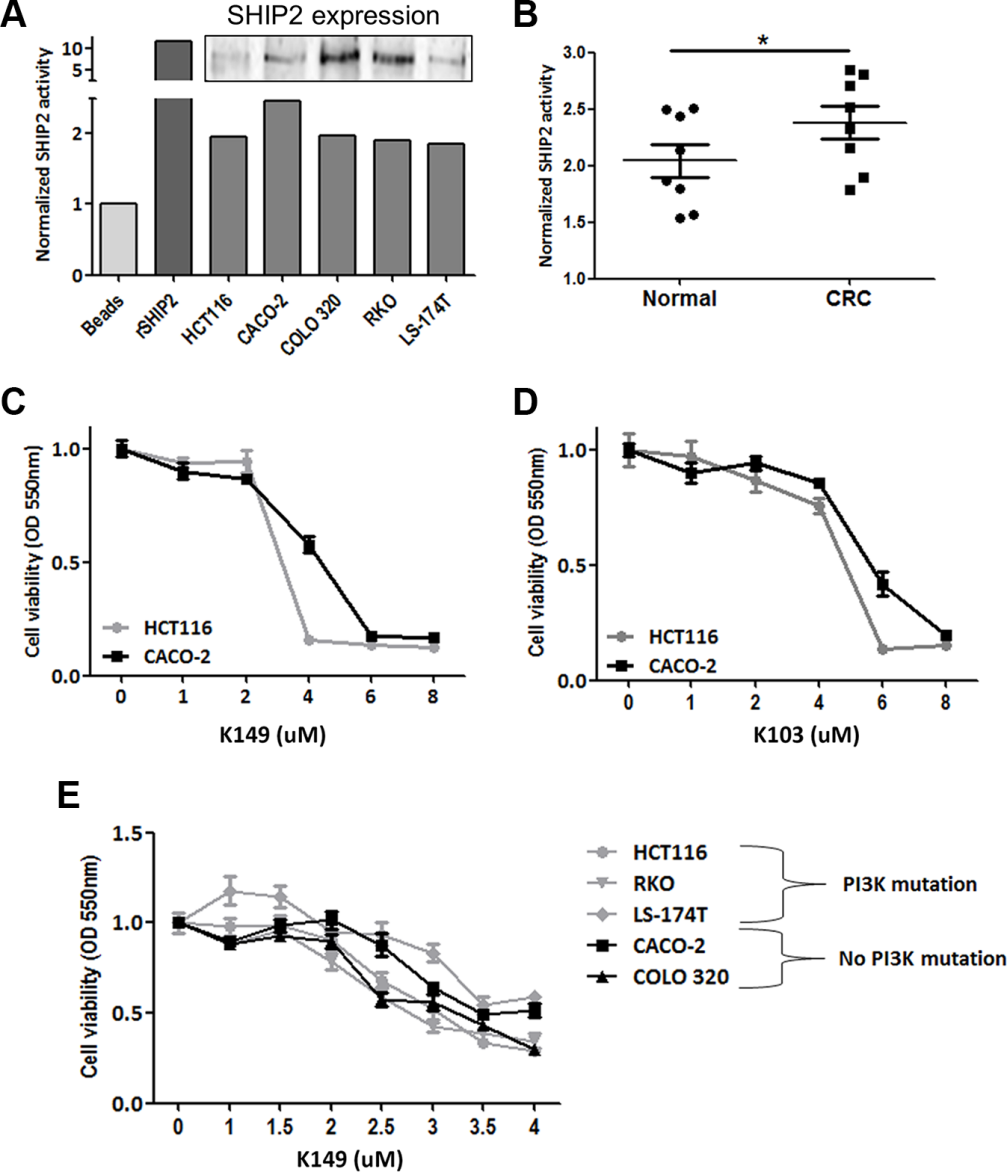
SHIP2 phosphatase activity is increased in CRC, and chemical SHIP2 activity inhibition results in CRC cells death (**A**) SHIP2 phosphatase assay shows reliable measurement of SHIP2 activity in CRC cell lines. Recombinant SHIP2 served as positive control and data were normalized to bead-only control. As SHIP2 precipitations were done under bead-saturating conditions, SHIP2 activity levels are a representation of intrinsic SHIP2 activity rather than different amounts of precipitated SHIP2. Insert shows SHIP2 protein levels in the cell line lysates. (**B**) SHIP2 activity was quantified in freshly frozen cancer and normal adjacent colonic tissue (*n* = 8), showing increased phosphatase activity in CRC. (**C**, **D**) Treatment of HCT116 and CACO-2 colorectal cancer cell lines with two different SHIP2 activity inhibitors (K149 and K103) results in a dose-dependent cell death. Since CACO-2 cells seem to be more resistant to SHIP2 inhibition, other cell lines with PI3K-mutations were tested. No relationship between SHIP2 inhibition and PI3K mutational status exists (**E**).

Since SHIP2 is an enzyme, the intrinsic phosphatase activity might be of even more importance than its total expression levels. Therefore, we set up an assay to investigate specific SHIP2 enzymatic activity in colon epithelial cells. This assay is based on the immunoprecipitation of SHIP2, after which the precipitated protein is incubated with its natural substrate PI(3,4,5)P_3_. Subsequently the amount of free phosphate was determined using Malachite Green. To test the validity of the assay, we quantified the SHIP2 activity in 5 different epithelial colorectal carcinoma cell lines, with recombinant SHIP2 as a positive control. As shown in Figure 3A, we were able to detect the intrinsic SHIP2 activity in all the cell lines. Next, we used the same assay to quantify the SHIP2 activity in freshly frozen resection specimens of colorectal carcinoma and paired normal adjacent tissue (*n* = 8). SHIP2 activity is significantly higher in colorectal cancer tissue in comparison to adjacent normal tissue (*p* < 0.05) (Figure 3B), demonstrating that not only is SHIP2 expression increased, this increase is functional in CRC tissue.

### SHIP2 inhibition reduces the amount of viable colorectal cancer cells, regardless of cell line specific mutations

As we found SHIP2 expression and activity to be upregulated in CRC as compared to non-cancerous tissue, we were interested in the effect of chemical SHIP2 activity inhibition on the cell viability of CRC cell lines. MTT assays were performed in 2 different epithelial colorectal cancer cell lines (HCT116 and CACO-2) with two different SHIP2 inhibitors (K103 (formerly 2PIQ) [13] and a novel analog that has potent SHIP2 inhibitory activity, K149 (Supplementary Figure 1A–1C). While both compounds induced a dose-dependent reduction in cell viability, both cell lines were more sensitive to the K149 inhibitor (Figure 3C–3D). Furthermore, both inhibitors induced cell death at lower concentrations in HCT116 cells than CACO-2 cells. Although both cell lines originate from human colorectal cancers, they have a very different mutational profile, with HCT116 cells harboring a H1047R mutation in *PIK3CA* gene, whereas CACO-2 cells do not. Since it has been previously shown that mutations in the *PIK3CA* gene confer differential sensitivity to drugs targeting the PI3K-PKB-pathway, we wondered whether this could also be true for the difference we observed with our SHIP2 inhibitors. However, upon comparison of 5 different cell lines (3 with *PIK3CA* mutation, 2 without) we did not observe a difference in sensitivity to SHIP2 inhibition based on *PIK3CA* mutational status (Figure 3E). This suggests that SHIP2 inhibitors might be a universal target for treatment of CRC, irrespective of genetic background.

### SHIP2 inhibition or knockdown reduces phosphorylated PKB levels, resulting in sensitivity to chemotherapeutics

**Figure 4:**
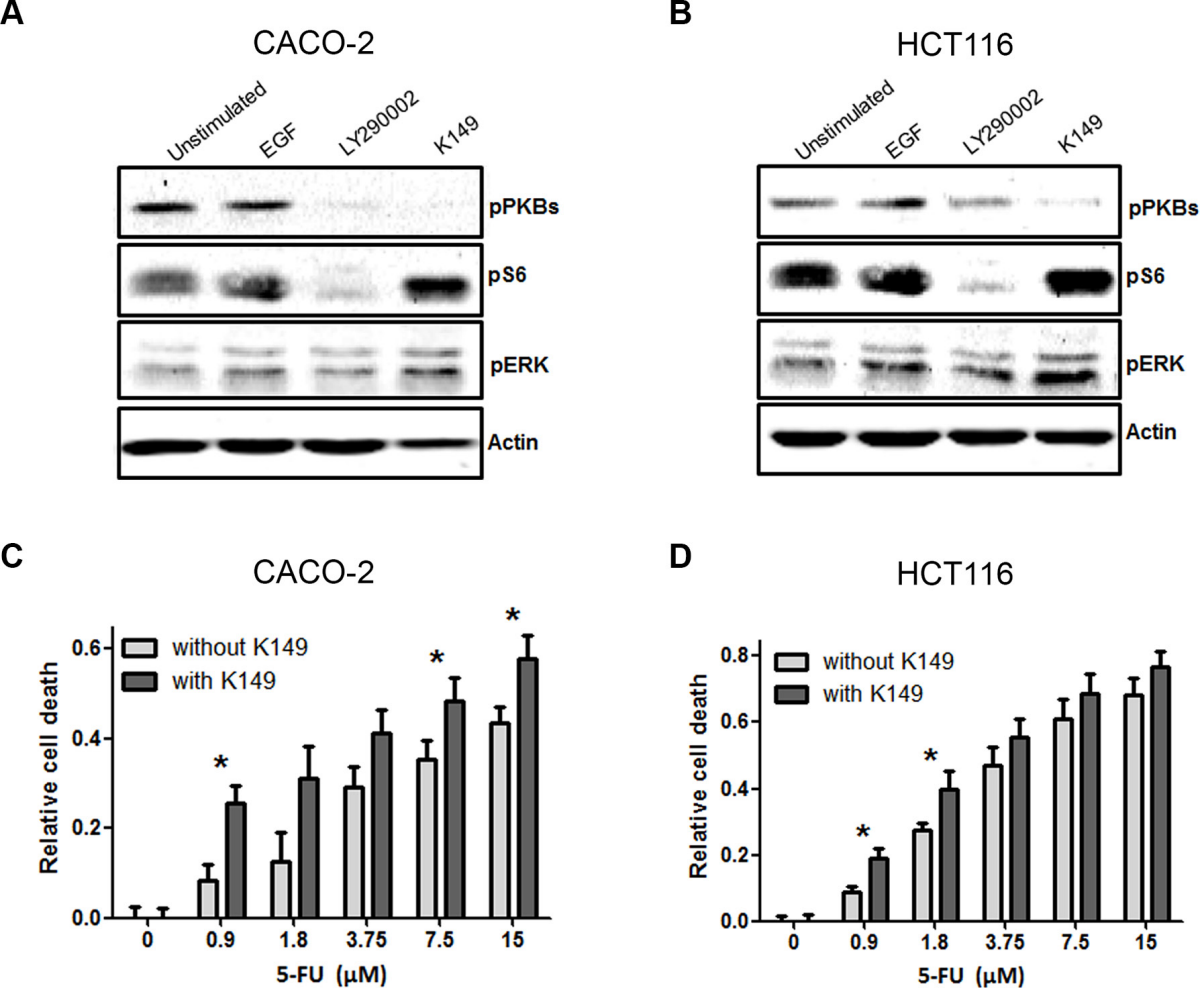
SHIP2 inhibition interferes with cancer signaling pathways, and sensitizes to 5-fluorouracil (5-FU) treatment (**A**, B) Treatment of CACO-2 and HCT116 cells with K149 results in decreased PKB phosphorylation, and increased pS6 phosphorylation. In contrast, control treatment with the PI3K inhibitor LY2940002 reduces both PKB phosphorylation and its downstream target pS6. ERK phosphorylation is affected by neither inhibitor. (**C**, **D**) 5-FU kills colorectal cancer cells in a dose dependent manner. In the presence of low concentrations of SHIP2 inhibitor, 5-FU-induced cell death is enhanced, in particular with low concentrations of 5-FU (*p < 0.05).

Since we observed a significant decrease in cell viability upon SHIP2 inhibition, we examined the effect of SHIP2 inhibition on the downstream signaling involved in processes like proliferation and cell survival (Figure 4A–4B). As expected, Epidermal Growth Factor (EGF) activates cellular signaling as shown by increased protein phosphorylation of PKB, ribosomal protein S6 and ERK. Furthermore, the PI3K inhibitor LY294002 inhibits activity of PKB and its downstream target ribosomal S6 protein. SHIP2 inhibition however, results in diminished phosphorylation of PKB, while phospho-S6 levels are drastically increased. ERK phosphorylation levels were not affected by either inhibitor, demonstrating their specificity. Together these data suggest that SHIP2 can affect mTOR signaling, independent of PKB. Since SHIP2 inhibition reduces PKB phosphorylation, we speculated that this reduced survival signal would sensitize cells to chemotherapeutic agents. When we treated intestinal epithelial cells with a low dose of the SHIP2 inhibitor K149 (2 uM), together with increasing concentrations of 5-FU, it was apparent that co-treatment with the SHIP2 inhibitor sensitizes CRC cells to the 5-FU treatment (Figure 4C–4D). This suggests that chemical SHIP2 inhibition would be a worthwhile addition to the standard chemotherapy regimen, requiring lower concentrations of chemotherapeutics to achieve the same amount of tumor cell killing.

### SHIP2 knockdown inhibits cell migration

**Figure 5:**
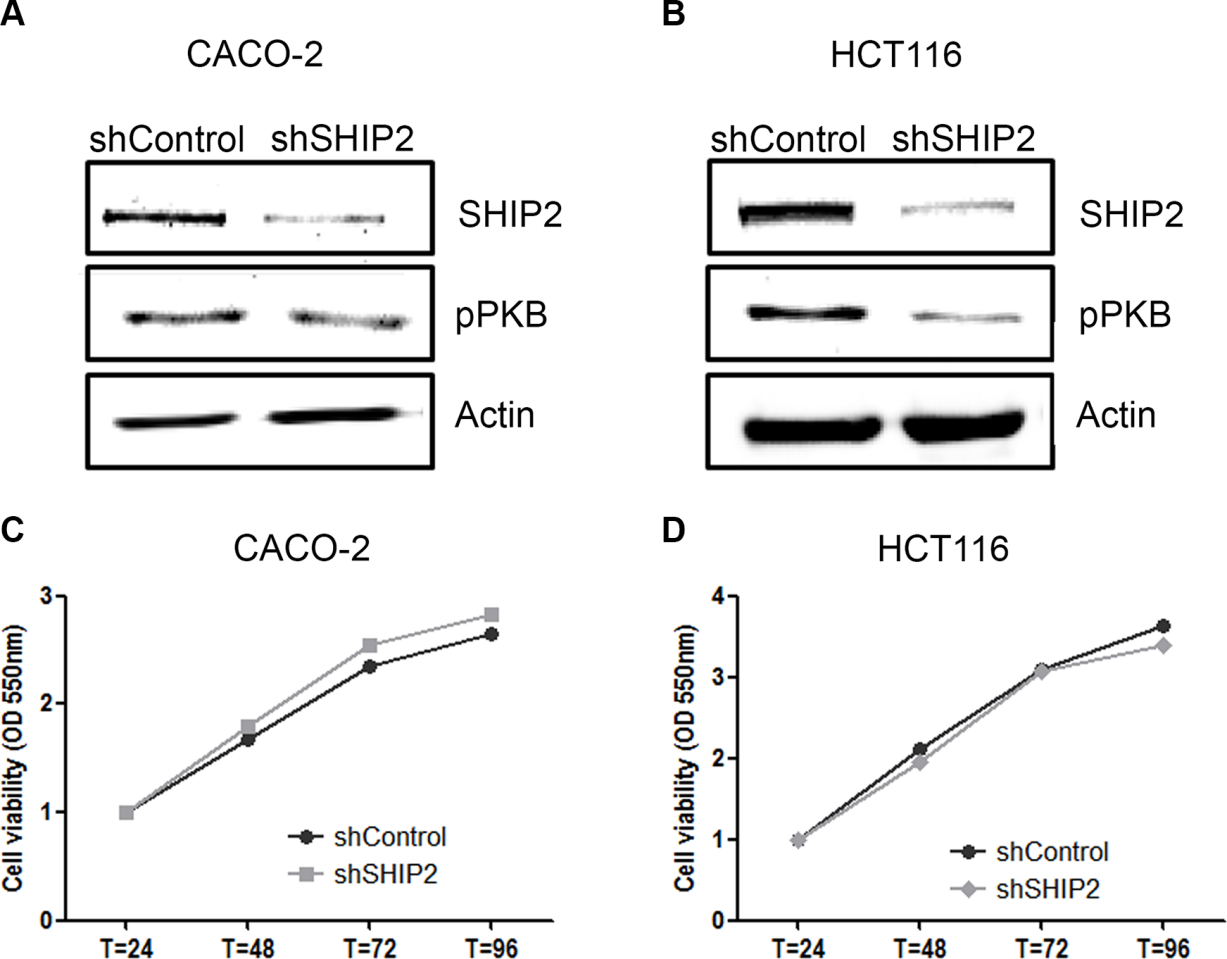
Stable knockdown of SHIP2 influences PKB signaling, without affecting cell proliferation (**A**, **B**) Lentiviral transduction of SHIP2 shRNA resulted in 80% reduction of SHIP2 expression levels, and reduces PKB phosphorylation as shon by western blot analysis. (**C**, **D**) Using MTT assays, cell proliferation was assessed, and no effect of SHIP2 knockdown was observed.

Metastasis is the major cause of cancer-related death, and thus it is important to study whether SHIP2 influences a cells' capability to migrate and form metastases. For this purpose, we stably transfected our CRC cell lines with shRNA against SHIP2 or non-target control. This resulted in SHIP2 knockdown cells, with approximately 80% reduction of SHIP2 levels, and a concomitant reduction in downstream PKB phosphorylation (Figure 5A–5B). Whereas treatment with chemical SHIP2 inhibitors results in CRC cell death, we did not observe any differences in proliferation between the SHIP2 knockdown and control cell lines (Figure 5C–5D). While this is perhaps not surprising - as SHIP2 knockdown was not complete, and the creation of stable cell lines would necessarily select for cells escaping cell death - this does make these knockdown cell lines excellent tools to study migratory behavior, especially since PKB is also known to influence cell migration [28].

**Figure 6:**
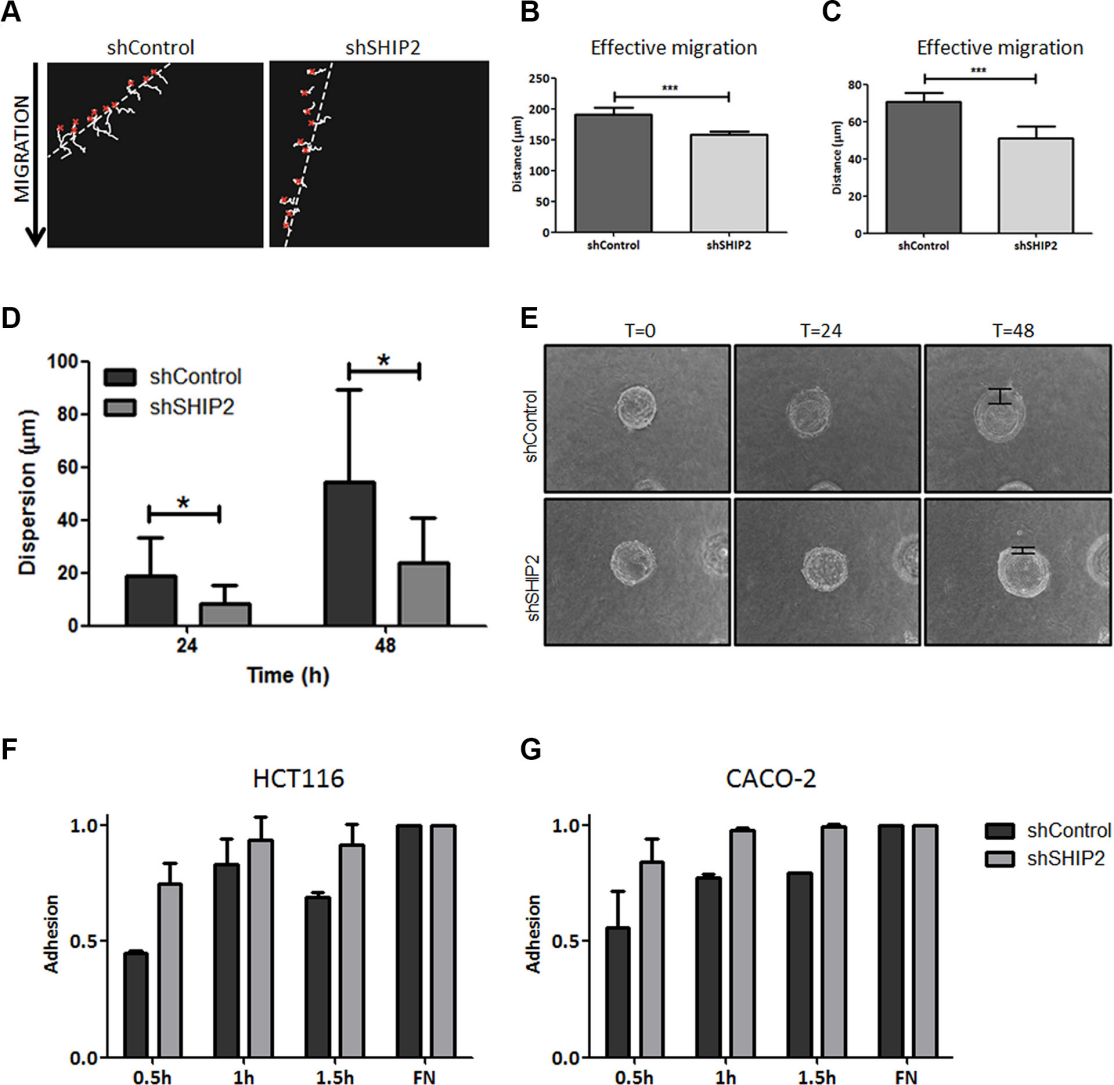
Modulation of SHIP2 expression affects migration, invasion, and adhesion in colorectal cancer cells (**A**) Cell migration was measured using a ring-barrier system. HCT116 cell migration on gelatin was tracked during 24 h, with locations being captured using time-lapse microscopy every 12min (x = start, line = cell track). (**B**, **C**) Quantification of migrated path indicates that the total migration and effective migration were significantly reduced in SHIP2 knockdown cells. (***p < 0.001). (**D**) Beads were coated with either HCT116 SHIP2 knockdown or control cells for 24 hours, and embedded in a collagen gel matrix. Cells were allowed to invade the collagen matrix, and pictures were taking at 0 h, 24 h, and 48 h (examples in E). The cell dispersion from the bead (arrow) into the collagen matrix was measured, and a trend towards reduced invasion was observed in SHIP2 knockdown cells. Data represents at least four beads. (*p* ≤ 0.05) (**F**, **G**) CRC cell adhesion was determined by MTT assay of adherent cells after indicated time points, with fibronectin (FN) coating serving as control. SHIP2 knockdown cells adhere more efficient to culture plates compared to non-target control.

2D ring-barrier migration assays using time-lapse microscopy were performed using SHIP2 knockdown and control cells. SHIP2 knockdown in HCT116 cells significantly inhibited total migration, as well as effective migration and velocity (*P* < 0.001) (Figure 6A–6C, Supplementary Video S1 and S2). Similarly, in CACO-2 cells, SHIP2 knockdown resulted in a slight decrease in total migration and velocity, with a great reduction in the effective migration (Supplementary Figure 2A–2C. Supplementary Video S3 and S4). Together these results suggest that SHIP2 is required for intestinal epithelial cell migration, and in particular directive migration.

### Reducing cellular SHIP2 results in reduced invasive capacity

In addition to cell migration, an equally important feature of metastasizing cancer cells is their capability to invade surrounding and distal tissues and matrices. Therefore, we performed 3D invasion assays for both HCT116 and CACO-2 non-target and SHIP2 knockdown cells, where cell migration is dependent on the cell capacity to break down surrounding matrigel. HCT116 control cells invade significantly further through collagen than their SHIP2 knockdown counterparts (time 48 h, *p* < 0.05) (Figure 6D–6E). Likewise, knockdown of SHIP2 in CACO-2 cells reduces early invasion compared to the parental cell line, although at later timepoints this effect was less pronounced (time 24 h, *p* > 0.05) (Supplementary Figure 2D–2E). Furthermore, we analyzed the anchorage-dependent growth of these cell lines using soft-agar colony growth assays. While we did not observe any differences in the number of colonies formed in the HCT116 cells, strikingly, CACO-2 cells downregulated for SHIP2 did not form any colonies at all (Supplementary Figure 2F–2G). Together these data suggest that SHIP2 indeed plays an important role in the invasive capacity of cancer cells.

### SHIP2 knockdown does not affect colon cancer cell adhesion

For malignant cells to escape their site of origin and start their invasive progress, modulation of cell adhesion is an important factor. Adhesion assays in HCT116 and CACO-2 non-target and knockdown cells were performed to study the role of SHIP2 in this process. HCT116 SHIP2 knockdown cells adhere more than HCT116 control cells, although this difference decreases over time. Likewise, SHIP2 reduction in CACO-2 cells slightly reduces adhesion as compared to the parental cell line. However, none of the differences are significant and thus the effect of SHIP2 on cell adhesion can be considered minimal (Figure 6F–6G).

## DISCUSSION

Phosphatases have long been regarded as undruggable tumor suppressor genes. However this dogma is currently changing as more data suggests that phosphatases do not only terminate cellular signaling, but can also promote signal transduction. In this study we identified the lipid phosphatase SHIP2 as an oncogene and potential treatment target in colorectal cancer. We show that the expression of this inositol phosphatase is increased in colorectal cancer tissue as compared to (adjacent) non-cancerous samples. This upregulation appears to be mediated on a transcriptional level, since mRNA levels of the SHIP2 encoding gene *INPPL1* are also increased in colorectal carcinoma samples. By comparing a large cohort of colorectal cancer patients, we found that patients with high SHIP2 expressing tumors have a poor outcome with earlier recurrence and reduced time to death. Together, these data suggest that SHIP2 increases the oncogenic potential of colonic epithelial cells. This fits well with previous reports, showing that SHIP2 expression is increased in breast cancer samples [29, 30] and that treatment of breast cancer cell lines with SHIP2 inhibitors results in cell death [13]. Other studies have also confirmed overexpression of SHIP2 in CRC, laryngeal squamous cell carcinoma and non-small lung cell cancer [31–33]. Importantly, we now show that the enzymatic activity of this phosphatase is also increased in colorectal cancer as compared to adjacent normal tissue, providing rationale for the treatment of this tumor type with SHIP2 activity inhibitors. Indeed, treatment of two colorectal cancer cell lines with a previously described SHIP2 inhibitor [34] and a novel analog resulted in a dose-dependent cell killing. Since we found HCT116 to be more sensitive to SHIP2 inhibition than CACO-2 cells, we hypothesized that this could be due to differences in their mutational background. It is known that tumors which harbor mutations in a particular pathway can become “addicted” to this pathway, and therefore become more sensitive to targeting of this signaling route [35]. Conversely, it has also been suggested that harboring a mutation in a particular gene renders tumors insensitive to inhibitors of signaling moieties downstream thereof [36]. While HCT116 cells harbor an activating mutation in the *PIK3CA* gene, CACO-2 cells do not. However upon comparison of 5 different CRC cell lines we could not relate the differences in sensitivity to SHIP2 inhibition to *PIK3CA* mutational status, suggesting that SHIP2 inhibition could be a target for treatment of CRC, irrespective of variation in their existing mutations. Likewise, in our TMA cohort, SHIP2 expression did not correlate to the mutational status of *PIK3CA*. However, although we have previously described selectivity of the K103 compound for SHIP1 and SHIP2 over the lipid phosphatase OCRL, we cannot formally exclude that these compounds have other, SHIP independent effects in the cell that may account for the lack of correlation to *PIK3CA* status of cells.

Our results demonstrating reduced PKB phosphorylation in CRC cells upon inhibition of SHIP2 are in line with our earlier results demonstrating reduced PKB phosphorylation in hematopoietic cells upon inhibition of the SHIP1 isoform [12]. Full PKB activation benefits from the presence of PI(3,4)P_2_, however, both PI(3,4,5)P_3_ and PI(3,4)P_2_ are required, providing a rationale for the previously coined ‘Two PIP Hypothesis’ which states that survival of cells is critically dependent on PIP_2_ : PIP_3_ ratios, where shifting the balance in either direction causes cell death [11, 13]. Indeed, both inhibition and over-activation of SHIP1 result in apoptosis of hematological multiple myeloma plasma cells [12, 37]. While ribosomal S6 protein is considered a direct downstream target of PKB activity through mTOR-p70S6 signaling, we observed that although PKB phosphorylation is decreased upon inhibition of SHIP2, S6 phosphorylation is enhanced. However, mTOR signaling is complex, with positive and negative feedback loops via the mTORC1 and mTORC2 signaling complexes, and cellular levels of PKB and S6 phosphorylation are thus not always unidirectional [38]. Alternatively, SHIP2 may affect the mTOR pathway independently from PKB. For instance, mTOR can be activated by Src kinase independent of PKB [39], and PI(3,4,5)P_3_, which is presumably increased upon SHIP2 knockdown, may activate other targets aside from PKB [40]. The cellular effect of the raised S6 phosphorylation levels in our model system is as yet unclear. Increased S6 phosphorylation is commonly associated with increased protein synthesis and autophagy inhibition, indicative of worse tumor phenotype [41–43]. However, we observe a reduced tumorigenic potential in our SHIP2 knockdown cells with increased S6 phosphorylation, suggesting that S6 is likely phosphorylated as a regulatory feedback mechanism. It should be noted however, that SHIP2 has phosphatase-independent docking functions, which may affect cell signaling independent of its effect on PKB and could account for some of its oncogenic potential [44].

While PKB can be targeted directly with small molecule inhibitors, SHIP2 modulation provides a novel molecular target to counteract this pathway. Recently it has been shown that using an allosteric AKT1/2/3 inhibitor in CRC patients does not result in the desired reduction in PKB phosphorylation levels, and therefore does not have clinical value [45]. Possibly these tumors would benefit from modification of SHIP2 activity, in order to reach the desired level of target inhibition. In addition, our data also suggest that treatment with SHIP2 inhibitors may sensitize CRC cells to other cytostatic treatments.

Previous reports have shown that SHIP2 can play a role in cellular adhesion, migration and cytoskeletal rearrangement. By interacting with filamin, p130cas, vinexin, or RhoA, SHIP2 localizes to the periphery of cells where it can modulate cytoskeletal organization and migration [46–51]. Furthermore, upon EGF-treatment, SHIP2 localizes to the lamellipodia where it induces an increased pool of PI(3,4)P_2_, which in turn is essential for podosomal formation and Ena/VASP recruitment [52, 53]. Together these data suggest SHIP2 can stimulate cytoskeletal rearrangement and cellular migration. In accordance with these results, we found that reducing SHIP2 expression in colorectal cancer cells results in decreased migration and increased cell adhesion. This effect is most apparent in the effective migration, suggesting that SHIP2 is an important factor for correct directional movement of cancer cells, and thereby crucial in the formation of cancer metastasis.

In summary, we found that the expression and intrinsic phosphatase activity of the lipid phosphatase SHIP2 is increased in human colorectal cancer, and that increased expression within a large cohort of CRC patient is correlated to a worse patient survival. SHIP2 functions as an oncogene, by enhancing cell migration and invasion, and reducing cell adhesion in colorectal cancer cells. Furthermore, treatment with a SHIP2 activity inhibitor results in dose-dependent cell death, and this inhibitor sensitizes CRC cells to chemotherapy treatment. Together, these data indicate that the SHIP2 phosphatase contributes to the malignant potential of colorectal cancer, providing a possible target in the fight against this devastating disease.

## MATERIALS AND METHODS

### Gene expression profiles

Using publicly available databases of RNA expression data (www.oncomine.com), *INPPL1* mRNA levels were analyzed. We aimed to identify all datasets with data available for *INPPL1* expression in a normal versus tumor analysis for colorectal cancer. This resulted in a comparison of 10 datasets.

### Patients and tumors on tissue micro array

Immunohistochemistry was initially performed on formalin fixed paraffin embedded (FFPE) colorectal tissue specimens from 9 low grade dysplasia (LGD) patients, 5 high grade dysplasia (HGD) patients and 11 adenocarcinoma (CRC) patients collected from the Erasmus Medical Center department of pathology. Inactive ulcerative colitis (UC) served as controls (*n* = 8). Staining was subsequently extended to a tissue microarray, consisting of a patient cohort of 470 colorectal cancer patients treated with surgery for their primary tumor in the Leiden University Medical Center (LUMC) between 1991 and 2001 [22]. Clinicopathological and follow-up data were collected retrospectively from hospital records and the hospitals' oncology database. This research was performed according to the code of conduct for responsible use. Patient records information was anonymized and deidentified prior to analysis according to national ethical guidelines (“Code for Proper Secondary Use of Human Tissue,” Dutch Federation of Medical Scientific Societies). Patients with multiple simultaneous colonic tumors were excluded from the analysis (*n* = 21). The entire study cohort consisted of 455 patients. Right-sided tumors were defined as those originating proximal to the splenic flexure and left-sided as those originating distal to the splenic flexure.

### Immunohistochemistry

FFPE tissue sections were immunohistologically stained for SHIP2 phosphatase. Briefly, 5 μm sections were deparaffinized in xylene and rinsed through graded alcohols (100% alcohols (18:1:1 100% ethanol: 100% methanol: 100% isopropanol), a 95% solution of the 100% alcohols, and a 80% solution of the 100% alcohols). Next, slides were rinsed several times with fresh deionized water, followed by another 5 minutes wash using fresh water. Antigen retrieval was performed by boiling the slides in 600 mL of 10 mM sodium citrate buffer, pH 6.0 in a glass 2 L-beaker for 15 minutes. Slides were cooled for 20 minutes and washed extensively in double-distilled H_2_O and PBS. Endogenous peroxides were blocked by soaking slides in a PBS/3% H_2_O_2_ solution for 10 minutes at room temperature. Subsequently, slides were rinsed in PBS and blocked by incubating in 10% goat serum in PBS at room temperature for 1h. Thereafter, tissue sections were incubated with primary antibody diluted in blocking buffer (1:100) overnight at 4°C. Next, slides were rinsed again in PBS for 5 minutes each wash. Rabbit envision (DAKO, Heverlee, Belgium) was used as secondary antibody. Slides were scored for the percentage of SHIP2 positive intestinal epithelial cells as well as intensity of the staining.

### Cell lines

Colorectal cancer cell lines HCT116, CACO-2, COLO 320, and RKO were cultured in Dulbecco's Modified Eagles Medium (DMEM, Lonza, Basel, Switzerland). Colorectal cancer cell line LS-174T was cultured in Roswell Park Memorial Institute (RPMI, Lonza, Basel, Switzerland) medium. All cell culture media were supplemented with 100 U/mL penicillin,100 mg/mL streptomycin (Life technologies, Bleiswijk, NL) and 10% Fecal Calf Serum (FCS, Sigma-Aldrich, St. Louis, USA). Cells were maintained at 37°C in a 5% CO_2_ humidified setting.

### Phosphatase assay

SHIP2 activity in HCT116, CACO-2, COLO 320, RKO and LS-174T cell lines was quantified by performing an immunoprecipitation based phosphatase assay [13]. Approximately 500.000 cells were transferred into 10 cm^2^ plates. After 24 h incubation, medium was removed and cells were washed with PBS. Cell were lysed on ice for two hours using 300 μL IP lysis buffer (20 mM Tris pH 7.5, 150 mM NaCl, 1 mM EDTA, 1 mM EGTA, and 1% Triton X100, 1mM PMSF and HALT protease inhibitors (ThermoFisher Scientific, Waltham, USA)). Lysates were cleared by centrifugation and supernatants were transferred into new 1.5 mL tubes. Subsequently, 3 μL SHIP2 antibody (Cell signaling technology, Leiden, the Netherlands) was added to each IP sample for antibody binding. Tubes were rotated overnight at 4°C. Protein AG beads (Pierce Protein A/G Plus Agarose) were used for purification of the formed protein-antibody complexes. Precipitates were washed 4 times with IP wash buffer and one time with TBS/10 mM MgCl_2_. After removing all liquid, precipitates were resuspended in 25 μL TBS/ 10 mM MgCl_2_, and 100 μM PI(3, 4, 5)P_3_ was added. Free phosphate produced through dephosphorylation of PI (3, 4, 5)P_3_ by SHIP2 present in the immunocomplexes was measured by addition of Malachite Green for 20 minutes, and read by a microplate reader (Model 680XR Bio-Rad) at 595 and 655 nm. Negative controls consisted of TBS/10 mM MgCl_2,_ TBS/10 mM MgCl_2_ with 3 μL PI(3, 4, 5)P_3_, and beads plus 3 μL PI(3, 4, 5)P_3_. Positive control consisted of TBS/10mM MgCl_2_ supplemented with 3 μL PI(3, 4, 5)P_3_ and 1 μg/ul recombinant SHIP2.

### Small molecule SHIP mnhibitors

The tryptamine based small molecule SHIP inhibitors 2PIQ (K103) and K149 were prepared as described in the supplement. Identity of the final products was established using ^1^H NMR, ^13^C NMR and mass spectroscopy. Purity of the final products was verified using ^1^H NMR, melting point and combustion analysis. Selective activity of the pan-SHIP inhibitor 2PIQ for SHIP1 and SHIP2 over the lipid phosphatase OCRL was demonstrated previously [13].

### Cell culture and transfections

To create stably transfected SHIP2 knockdown cells, HEK293T cells were transfected with 2 μg of shRNA with sequence CCGGATTCTGTGGAAATCCTA (Sigma-Aldrich, St. Louis, USA), together with lentiviral vectors in a 6 well plate. Non-targeting shRNA was used as a control. At 48 h and 72 h virus-containing medium was collected and passed through a 0.45 μM filter. Next, colorectal cancer cell lines HCT116 and CACO-2 cells were incubated with the virus-containing medium for 48 h. Subsequently, cells were selected by 2 μg/mL Puromycin (Sigma-Aldrich, St. Louis, USA), and knockdown was confirmed by Western blotting for SHIP2.

### Cell viability assay

Cell viability was assessed using MTT assays. Cells were incubated with different concentrations of SHIP2 inhibitors, and/or chemotherapeutic agent 5-FU. 24 h, 48 h, 72 h, and 96 h after incubation cells were incubated with 5 mM MTT (3-(4,5-Dimethylthiazol-2-yl)-2,5-diphenyltetrazolium bromide) for 3h and colorimetric changes were measured using a microplate reader (Model 680XR Bio-Rad) at 490 and 595 nm.

### Western blotting

HCT116 and CACO-2 cells were serum starved by incubating for 2h, after which cells were stimulated for 15 minutes with EGF (10ng/mL), or stimulated for 1 h with LY2940002 (20 μM) or SHIP2 inhibitor K149 (10 μM). Subsequently, cells were washed with PBS and lysed on ice in 300 μL 2× concentrated Laemmli buffer (100 mM Tris–HCl (pH 6.8), 200 mM dithiothreitol, 4% SDS, 0.1% bromophenol blue, 20% glycerol, and 2% DTT) and boiled for 5 minutes at 95°C. Cell extracts were resolved by SDS–PAGE and transferred to polyvinylidene difluoride membranes (Merck chemicals BV, Amsterdam, the Netherlands). Membranes were blocked in 50% odyssey blocking buffer (LI-COR Biosciences, Lincoln, NE) in PBS/0.05% Tween-20 and incubated overnight at 4°C with primary antibody. After washing in PBS-T, membranes were incubated with IRDye^®^ antibodies (LI-COR Biosciences, Lincoln, NE) for 1h. Detection was performed using Odyssey reader and analyzed using manufacturers software.

### 2D migration assay

Cell migration was analyzed using the “ring barrier system” as described previously [23]. First, coverslips were coated with fibronectin (10 mg/mL) and incubated in a humidified setting for 1h, prior to cell seeding. Thereafter, a circular sterile migration barrier was inserted into the chamber. This barrier prevents cell growth in the center of the coverslip. Subsequently, cells were incubated for 24 h, resulting in a confluent monolayer in the periphery and a cell-free area in the center on the well. Next, the circular migration barrier was removed. Migration of the cells into the open area was tracked for 24 h using time-lapse microscopy (Carl Zeiss B.V., Sliedrecht, Netherlands), and several measurements were performed. All migration assays were conducted in presence of 10% FCS.

### 3D migration assay

Cytodex-3 microcarrier beads (Sigma–Aldrich) were mixed with 5 × 10^5^ CACO-2 and HCT116 knockdown and control cell suspensions, at a density of 40 cells per bead, and incubated at 37°C for 6 h with gentle mixing. The bead suspension was transferred to a 25 cm^2^ tissue culture flask and incubated for 48 h to ensure complete coating of beads and to remove unattached cells. Coated beads were embedded in 1.6 mg/ml collagen gel (collagen: modified Eagle's medium:7.5% w/v NaHCO3 in the ratio 8:1:1) in a 24-well plate such that each well had approximately 150 beads. Plates were incubated at 37°C for 2 h for the beads to settle in the gel and the polymerized gels were covered with 500 μl DMEM, 10% FBS, 1% p/s. Cell dispersion was measured as the maximum migrated distance from the surface of the bead into the collagen gel. All measurements were performed using AxioVision 4.5 software and assays were performed three times in duplicate. Two-way analysis of variance was performed to calculate *P*-values.

### Adhesion assay

Adhesion assay was performed by loading 50.000 cells into 96 well plates. Cells were allowed to adhere to the well surface, coated with either fibronectin (Athena Enzyme Systems^TM^) or without coating, for different time points, incubated at humidified setting. After 30 min, 1h and 1.5 hours non-adherent cells were washed away. After 1.5 hours, MTT was added to the plate in order to quantify the amount of adhered cells. Cells coated with fibronectin for 1.5 h served as control.

### Statistics

All the graphs and the statistical analyses for the *in vitro* experiments were compared using a students' *T-test* and performed using the Graphpad Prism 5.0 software package for Windows. A two-tailed *p-value* < 0.05 was accepted as statistically significant. Images were composed using Adobe Photoshop CS6.

## SUPPLEMENTARY MATERIALS FIGURE, TABLES AND VIDEO











## References

[1] Siegel R, Desantis C, Jemal A (2014). Colorectal cancer statistics. CA Cancer J Clin.

[2] Van Cutsem E, Köhne CH, Hitre E, Zaluski J, Chang Chien CR, Makhson A, D'Haens G, Pintér T, Lim R, Bodoky G, Roh JK, Folprecht G, Ruff P (2009). Cetuximab and chemotherapy as initial treatment for metastatic colorectal cancer. N Engl J Med.

[3] Folprecht G, Gruenberger T, Bechstein WO, Raab HR, Lordick F, Hartmann JT, Lang H, Frilling A, Stoehlmacher J, Weitz J, Konopke R, Stroszczynski C, Liersch T (2010). Tumour response and secondary resectability of colorectal liver metastases following neoadjuvant chemotherapy with cetuximab: the CELIM randomised phase 2 trial. Lancet Oncol.

[4] Douillard JY, Siena S, Cassidy J, Tabernero J, Burkes R, Barugel M, Humblet Y, Bodoky G, Cunningham D, Jassem J, Rivera F, Kocákova I, Ruff P (2014). Final results from PRIME: randomized phase III study of panitumumab with FOLFOX4 for first-line treatment of metastatic colorectal cancer. Ann Oncol.

[5] Seshacharyulu P, Ponnusamy MP, Haridas D, Jain M, Ganti AK, Batra SK (2012). Targeting the EGFR signaling pathway in cancer therapy. Expert Opinion on Therapeutic Targets.

[6] Han CB, Li F, Ma JT, Zou HW (2012). Concordant KRAS Mutations in Primary and Metastatic Colorectal Cancer Tissue Specimens: A Meta-Analysis and Systematic Review. Cancer Investigation.

[7] Lièvre A, Bachet JB, Le Corre D, Boige V, Landi B, Emile JF, Côté JF, Tomasic G, Penna C, Ducreux M, Rougier P, Penault-Llorca F, Laurent-Puig (2006). KRAS mutation status is predictive of response to cetuximab therapy in colorectal cancer. Cancer Res.

[8] Grothey A, Van Cutsem E, Sobrero A, Siena S, Falcone A, Ychou M, Humblet Y, Bouché O, Mineur L, Barone C, Adenis A, Tabernero J, Yoshino T (2013). Regorafenib monotherapy for previously treated metastatic colorectal cancer (CORRECT): an international, multicentre, randomised, placebo-controlled, phase 3 trial. Lancet.

[9] Wong KK, Engelman JA, Cantley LC (2010). Targeting the PI3K signaling pathway in cancer. Current Opinion in Genetics and Development.

[10] Hoekstra E, Peppelenbosch MP, Fuhler GM (2012). The role of protein tyrosine phosphatases in colorectal cancer. Biochim Biophys Acta.

[11] Kerr WG (2011). Inhibitor and activator: dual functions for SHIP in immunity and cancer. AnnNYAcadSci [Internet].

[12] Brooks R, Fuhler GM, Iyer S, Smith MJ, Park MY, Paraiso KHT, Engelman RW, Kerr WG (2010). SHIP1 inhibition increases immunoregulatory capacity and triggers apoptosis of hematopoietic cancer cells. J Immunol.

[13] Fuhler GM, Brooks R, Toms B, Iyer S, Gengo EA, Park MY, Gumbleton M, Viernes DR, Chisholm JD, Kerr WG (2011). Therapeutic potential of SHIP1 and SHIP2 inhibition in cancer cells. Mol Med.

[14] Pesesse X, Deleu S, De Smedt F, Drayer L, Erneux C (1997). Identification of a second SH2-domain-containing protein closely related to the phosphatidylinositol polyphosphate 5-phosphatase SHIP. Biochem Biophys Res Commun.

[15] Bunney TD, Katan M (2010). Phosphoinositide signalling in cancer: beyond PI3K, PTEN. Nat Rev Cancer.

[16] Yamada KM, Araki M (2001). Tumor suppressor PTEN: modulator of cell signaling, growth, migration and apoptosis. J Cell Sci.

[17] Scheid MP, Huber M, Damen JE, Hughes M, Kang V, Neilsen P, Prestwich GD, Krystal G, Duronio V (2002). Phosphatidylinositol (3,4,5)P3 is essential but not sufficient for protein kinase B (PKB) activation; phosphatidylinositol (3,4)P2 is required for PKB phosphorylation at Ser-473: studies using cells from SH2-containing inositol-5-phosphatase knockout mice. J Biol Chem.

[18] Elong Edimo W, Schurmans S, Roger PP, Erneux C (2014). SHIP2 signaling in normal and pathological situations: Its impact on cell proliferation. Advances in Biological Regulation.

[19] Prasad NK, Tandon M, Badve S, Snyder PW, Nakshatri H (2008). Phosphoinositol phosphatase SHIP2 promotes cancer development and metastasis coupled with alterations in EGF receptor turnover. Carcinogenesis.

[20] Hodgson MC, Shao L, Frolov A, Li R, Peterson LE, Ayala G, Ittmann MM, Weigel NL, Agoulnik IU (2011). Decreased expression and androgen regulation of the tumor suppressor gene INPP4B in prostate cancer. Cancer Res.

[21] Agoulnik IU, Hodgson MC, Bowden WA, Ittmann MM (2011). INPP4B: the new kid on the PI3K block. Oncotarget.

[22] Zeestraten ECM, Reimers MS, Saadatmand S, Dekker JWT, Liefers GJ, van den Elsen PJ, van de Velde CJH, Kuppen PJK (2014). Combined analysis of HLA class I, HLA-E, HLA-G predicts prognosis in colon cancer patients. Br J Cancer.

[23] Hoekstra E, Kodach LL, Das AM, Ruela-de-Sousa RR, Ferreira CV, Hardwick JC, van der Woude CJ, Peppelenbosch MP, ten Hagen TLM, Fuhler GM (2015). Low molecular weight protein tyrosine phosphatase (LMWPTP) upregulation mediates malignant potential in colorectal cancer. Oncotarget.

[24] Hong Y, Downey T, Eu KW, Koh PK, Cheah PY (2010). A “metastasis-prone” signature for early-stage mismatch-repair proficient sporadic colorectal cancer patients and its implications for possible therapeutics. Clin Exp Metastasis.

[25] Kaiser S, Park YK, Franklin JL, Halberg RB, Yu M, Jessen WJ, Freudenberg J, Chen X, Haigis K, Jegga AG, Kong S, Sakthivel B, Xu H (2007). Transcriptional recapitulation and subversion of embryonic colon development by mouse colon tumor models and human colon cancer. Genome Biol.

[26] Skrzypczak M, Goryca K, Rubel T, Paziewska A, Mikula M, Jarosz D, Pachlewski J, Oledzki J, Ostrowsk J (2010). Modeling oncogenic signaling in colon tumors by multidirectional analyses of microarray data directed for maximization of analytical reliability. PLoS One.

[27] Budczies J, Klauschen F, Sinn BV, Gyorffy B, Schmitt WD, Darb-Esfahani S, Denkert C (2012). Cutoff Finder: A Comprehensive and Straightforward Web Application Enabling Rapid Biomarker Cutoff Optimization. PLoS One.

[28] Fuhler GM, Drayer AL, Olthof SGM, Schuringa JJ, Coffer PJ, Vellenga E (2008). Reduced activation of protein kinase B, Rac, and F-actin polymerization contributes to an impairment of stromal cell-derived factor-1-induced migration of CD34+ cells from patients with myelodysplasia. Blood.

[29] Prasad NK, Tandon M, Handa A, Moore GE, Babbs CF, Snyder PW, Bose S (2008). High expression of obesity-linked phosphatase SHIP2 in invasive breast cancer correlates with reduced disease-free survival. Tumour Biol.

[30] Fu CH, Lin RJ, Yu J, Chang WW, Liao GS, Chang WY, Tseng LM, Tsai YF, Yu JC, Yu AL (2014). A novel oncogenic role of inositol phosphatase SHIP2 in ER-negative breast cancer stem cells: involvement of JNK/vimentin activation. Stem Cells.

[31] Zhou X, Liu Y TG (2011). Prognostic value of elevated SHIP2 expression in laryngeal squamous cell carcinoma. Arch Med Res.

[32] Yang J, Fu M, Ding Y, Weng Y, Fan W, Pu X (2014). High SHIP2 expression indicates poor survival in colorectal cancer. Dis Markers.

[33] Yang J, Fu M, Ding Y, Weng Y, Fan W, Pu X, Ge Z, Zhan F, Ni H, Zhang W, Jin F, Xu N, Li J (2013). Elevated expression of SHIP2 correlates with poor prognosis in non-small cell lung cancer. Int J Clin Exp Pathol.

[34] Viernes DR, Choi LB, Kerr WG, Chisholm JD (2014). Discovery and Development of Small Molecule SHIP Phosphatase Modulators. Med Res Rev.

[35] Torti D, Trusolino L (2011). Oncogene addiction as a foundational rationale for targeted anti-cancer therapy: Promises and perils. EMBO Mol Med.

[36] Ellis LM, Hicklin DJ (2009). Resistance to targeted therapies: Refining anticancer therapy in the era of molecular oncology. Clinical Cancer Research.

[37] Kennah M, Yau TY, Nodwell M, Krystal G, Andersen RJ, Ong CJ, Mui ALF (2009). Activation of SHIP via a small molecule agonist kills multiple myeloma cells. Exp Hematol.

[38] Fuhler GM, Tyl MR, Olthof SGM, Lyndsay Drayer A, Blom N, Vellenga E (2009). Distinct roles of the mTOR components Rictor and Raptor in MO7e megakaryocytic cells. Eur J Haematol.

[39] Vojtechová M, Turecková J, Kucerová D, Sloncová E, Vachtenheim J, Tuhácková Z (2008). Regulation of mTORC1 signaling by Src kinase activity is Akt1-independent in RSV-transformed cells. Neoplasia.

[40] Rameh L, Cantley LC (1999). The role of phosphoinositide 3-kinase lipid products in cell function. J Biol Chem.

[41] Annovazzi L, Mellai M, Caldera V, Valente G, Tessitore L, Schiffer D (2009). mTOR, S6 and AKT expression in relation to proliferation and apoptosis/autophagy in glioma. Anticancer Res.

[42] Hay N, Sonenberg N (2004). Upstream and downstream of mTOR. Genes and Development.

[43] Nozawa H, Watanabe T, Nagawa H (2007). Phosphorylation of ribosomal p70 S6 kinase and rapamycin sensitivity in human colorectal cancer. Cancer Lett.

[44] Erneux C, Edimo WE, Deneubourg L, Pirson I (2011). SHIP2 multiple functions: a balance between a negative control of PtdIns(3,4,5)P_3_ level, a positive control of PtdIns(3,4)P_2_ production, and intrinsic docking properties. J Cell Biochem.

[45] Do K, Speranza G, Bishop R, Khin S, Rubinstein L, Kinders RJ, Datiles M, Eugeni M, Lam MH, Doyle LA, Doroshow JH, Kummar S (2015). Biomarker-driven phase 2 study of MK-2206 and selumetinib (AZD6244, ARRY-142886) in patients with colorectal cancer. Invest New Drugs.

[46] Prasad N, Topping RS, Decker SJ (2001). SH2-containing inositol 5′-phosphatase SHIP2 associates with the p130(Cas) adapter protein and regulates cellular adhesion and spreading. Mol Cell Biol.

[47] Prasad NK, Decker SJ (2005). SH2-containing 5′-inositol phosphatase, SHIP2, regulates cytoskeleton organization and ligand-dependent down-regulation of the epidermal growth factor receptor. J Biol Chem.

[48] Prasad NK (2009). SHIP2 phosphoinositol phosphatase positively regulates EGFR-Akt pathway, CXCR4 expression, and cell migration in MDA-MB-231 breast cancer cells. Int J Oncol.

[49] Paternotte N, Zhang J, Vandenbroere I, Backers K, Blero D, Kioka N, Vanderwinden JM, Pirson I, Erneux C, Paternotte N, Zhang J, Vandenbroere I, Backers K (2005). SHIP2 interaction with the cytoskeletal protein Vinexin. FEBS J.

[50] Onnockx S, De Schutter J, Blockmans M, Xie J, Jacobs C, Vanderwinden JM, Erneux C, Pirson I (2008). The association between the SH2-containing inositol polyphosphate 5-phosphatase 2 (SHIP2) and the adaptor protein APS has an impact on biochemical properties of both partners. J Cell Physiol.

[51] Kato K, Yazawa T, Taki K, Mori K, Wang S, Nishioka T, Hamaguchi T, Itoh T, Takenawa T, Kataoka C, Matsuura Y, Amano M, Murohara T (2012). The inositol 5-phosphatase SHIP2 is an effector of RhoA and is involved in cell polarity and migration. Molecular Biology of the Cell.

[52] Oikawa T, Takenawa T (2009). PtdIns(3,4)P2 instigates focal adhesions to generate podosomes. Cell Adhesion and Migration.

[53] Yoshinaga S, Ohkubo T, Sasaki S, Nuriya M, Ogawa Y, Yasui M, Tabata H, Nakajima K (2012). A Phosphatidylinositol Lipids System, Lamellipodin, and Ena/VASP Regulate Dynamic Morphology of Multipolar Migrating Cells in the Developing Cerebral Cortex. Journal of Neuroscience.

