# Supplementary material for: Lipid phosphatase SHIP2 functions as oncogene in colorectal cancer by regulating PKB activation
Source: Oncotarget. 2016 Sep 28;7(45):73525–40. doi: 10.18632/oncotarget.12321 (PMC5341996; doi:10.18632/oncotarget.12321)
Supplement: Supplementary file 1 [file oncotarget-07-73525-s001.pdf]

Lipid phosphatase SHIP2 functions as oncogene in colorectal cancer by regulating PKB activation

Supplementary Materials

SUPPLEMENTARY MATERIALS AND METHODS

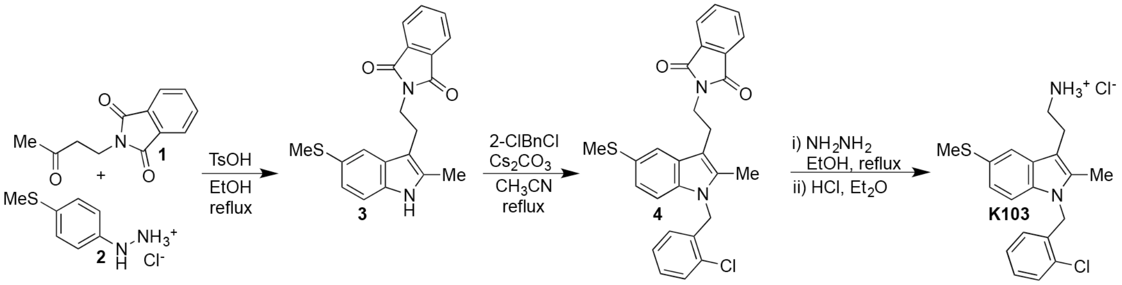

Preparation and characterization of the tryptamine based small molecule SHIP inhibitors 2PIQ (K103) and K149.

2-(2-(2-Methyl-5-methylthio)-1H-indol-3-yl)ethyl) isoindoline-1,3-dione, 3

N-(4-Oxopentyl) phthalimide 1[1] (1.09 g, 4.72 mmol) and 4-methylthio phenyl hydrazine hydrochloride 2[2] (1.00 g, 5.24 mmol) were dissolved in 20 mL of ethanol. TsOH•H2O (3.97 g, 20.84 mmol) was then added. The reaction mixture was heated to reflux for approximately 16 h. The reaction mixture was then cooled to room temperature and poured into 60 mL of 1 M NaOH. The mixture was then extracted with dichloromethane (3 × 20 mL). The organic extracts were combined, dried over Na2SO4, filtered and concentrated. Purification using silica gel chromatography (40% ethyl acetate/hexanes) provided 1.39 g (84%) of indole 3 as a yellow foam.

mp = 181–184 °C; TLC R_f = 0.40 (30% ethyl acetate in hexanes); IR (thin film) 3435, 2927, 1772, 1712, 1469, 1396, 651 cm^{-1}; ^1H NMR (400 MHz, $CDCl_3$) δ 7.87–7.82 (m, 2H), 7.77 (br s, 1H), 7.74–7.69 (m, 2H), 7.62 (s, 1H), 7.19 (dd, *J* = 8.0, 0.4 Hz, 1H), 7.12 (dd, *J* = 8.4, 1.6 Hz, 1H), 3.93–3.89 (m, 2H), 3.08–3.04 (m, 2H), 2.52 (s, 3H), 2.43 (s, 3H); ^{13}C NMR (100 MHz, $CDCl_3$) δ 167.3, 132.83, 132.81, 131.7, 131.1, 128.4, 126.5, 122.1, 122.0, 117.5, 109.7, 106.8, 37.2, 22.3, 17.7, 10.5.

2-[2-(2-Methyl-5-thiomethyl-N-(o-chloro benzyl indol-3-yl)-ethyl]-1H-isoindole-1,3-dione-(2H), 4

Indole 3 (103 mg, 0.293 mmol) was dissolved in 3 mL of acetonitrile. Cesium carbonate (477 mg, 1.465 mmol) was added followed by 2-chlorobenzyl chloride (100 μL, 0.791 mmol). The reaction mixture was then heated to reflux for 24 h. The suspension was then allowed to cool to room temperature, quenched with brine (20 mL), the organic layer separated, and the aqueous layer extracted with DCM (3 × 10 mL). The combined organic extracts were dried with Na_2SO_4, filtered, and concentrated. Purification by silica gel chromatography (DCM) provided the N-benzylated indole 4 (91 mg, 65%) as a yellow foam.

TLC R_f = 0.68 (30% ethyl acetate in hexanes); IR (thin film) 3001, 2990, 1716, 1650, 1363, 1222, 1093, 760 cm^{-1}; ^1H NMR (400 MHz, $CDCl_3$) δ 7.80–7.76 (m, 2H), 7.67–7.63 (m, 3H), 7.37 (dd, *J* = 8.0, 0.8 Hz, 1H), 7.15 (td, *J* = 7.6, 1.2 Hz, 1H), 7.07–6.97 (m, 3H), 6.18 (dd, *J* = 8.0, 1.2 Hz, 1H), 5.27 (s, 2H), 3.92 (t, *J* = 7.2 Hz, 2H), 3.11 (t, *J* = 7.6 Hz, 2H), 2.47 (s, 3H), 2.25 (s, 3H); ^{13}C NMR (100 MHz, $CDCl_3$) δ 168.3, 135.1, 135.0, 134.3, 133.9, 132.1, 131.8, 129.3, 128.8, 128.5, 127.8, 127.4, 126.9, 123.1, 123.0, 118.5, 109.5, 108.0, 44.4, 38.4, 23.6, 18.6, 10.0.

2-[1-(2-Chlorobenzyl)-2-methyl-5-(methylmercapto)-1H-3-yl] ethanaminium chloride, K103

Phthalimide 4 (1.168 g, 2.46 mmol) was dissolved in 20 mL methanol. Tetrahydrofuran (10 mL) was added to form a solution. Hydrazine hydrate (85%, 480 μL, 9.84 mmol) was then added and the reaction mixture was heated to reflux. After 3 h the reaction mixture was cooled to room temperature and concentrated. The resulting residue was purified by silica gel chromatography (90% dichloromethane: 9% methanol: 1% ammonium hydroxide). The resulting free amine was then dissolved in 5 mL of diethyl ether. A solution of hydrogen chloride in diethyl ether (2 M, 5.82 mmol, 2.9 mL) was then

added, and an off-white precipitate formed. The reaction mixture was allowed to stand for 20 minutes and then concentrated. Recrystallization from methanol gave 587 mg (53%) of K103 as an off-white powder.

K103. mp = 191–193 °C. IR (thin film) 3400, 2908, 2942, 2900, 1551, 1375, 1242 cm^{-1}; ^1H NMR (400 MHz, DMSO-d_6) δ 7.57–7.48 (m, 5H), 7.31–7.25 (m, 2H), 7.16 (td, J = 7.6, 1.2 Hz, 1H), 7.05 (dd, J = 8.4, 1.6 Hz, 1H), 6.20 (dd, J = 7.6, 1.2 Hz, 1H), 5.43 (s, 2H), 3.02–2.92 (m, 4H), 2.50 (s, 3H), 2.26 (s, 3H); ^{13}C NMR (100 MHz, DMSO-d_6) δ 140.5, 140.2, 139.9, 136.3, 134.6, 134.1, 133.4, 132.8, 132.3, 132.0, 127.0, 122.7, 115.3, 112.0, 49.1, 45.0, 28.0, 22.6, 15.0. HRMS (ESI$^+$) *m/z* calcd for $C_{19}H_{19}ClNS$ [M-NH3]$^+$: 328.0921. Found: 328.0912. Anal calcd for $C_{19}H_{22}Cl2N2S$: C, 59.84; H, 5.81; N, 7.35. Found: C, 59.86; H, 5.76; N, 7.26.

2-[1-(2,4-Dichlorobenzyl)-2-methyl-5-(methylmercapto)-1H-3-yl] ethanaminium chloride, K149

Phthalimide 5 (5.22 g, 10.25 mmol) was dissolved in 102 mL ethanol. Hydrazine monohydrate (2.50 mL, 51.27 mmol) was added and the resulting mixture was heated to reflux for 18 h. The reaction mixture was cooled to room temperature and a precipitate formed which was removed by filtration. The filtrate was concentrated *in vacuo* and then purified using silica gel chromatography (1% triethylamine/1.5% methanol/97.5% DCM) to give the free amine as a white powder. The amine was then dissolved in a mixture of diethyl ether and methanol (10:1) and HCl gas was bubbled through the mixture for ~20 min. The resulting precipitate was filtered and washed with diethyl ether to afford 1.05 g (25%) of K149 as a tan powder.

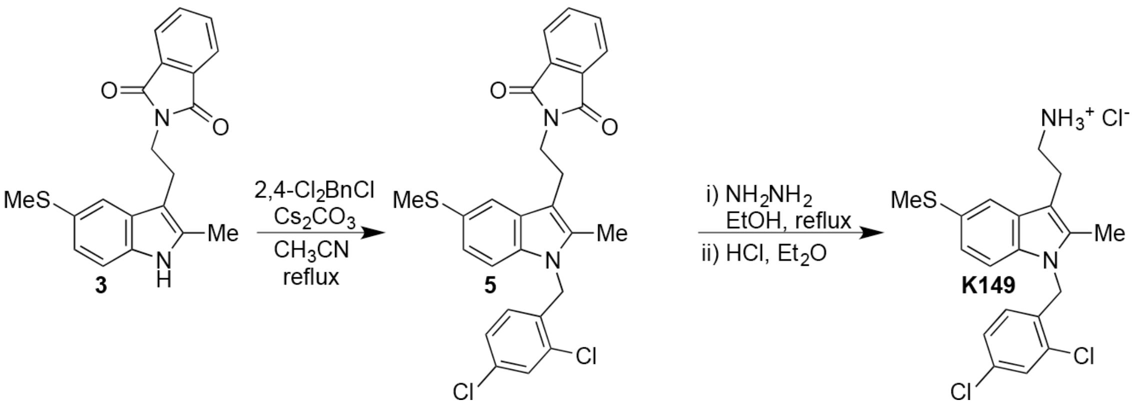

2-[2-(2-Methyl-5-thiomethyl-N-(2,4-dichloro benzyl indol-3-yl)-ethyl]-1H-isoindole-1,3-dione-(2H), 5

Tryptamine 3 (7.88 g, 22.51 mmol) was dissolved in 90 mL acetonitrile. 2,4-Dichlorobenzylchloride (6.3 mL, 45.02 mmol) and cesium carbonate (36.5 g, 112.57 mmol) were then added. The resulting mixture was heated to reflux for 2 hrs. The reaction mixture was cooled to room temperature and then filtered. The filtrate obtained was concentrated under reduced pressure to afford the crude product, which was then purified by silica gel column chromatography using 15% ethyl acetate/85% hexanes to yield 5.22 g (46%) of protected tryptamine 5 as a yellow foam.

IR (thin film) 3088, 2921, 2860, 1770, 1710 cm^{-1}; ^1H NMR (400 MHz, $CDCl_3$) δ 7.80–7.77 (m, 2H), 7.69–7.67 (m, 2H), 7.60 (d, J = 1.6 Hz, 1H), 7.41 (d, J = 2.0 Hz, 1H), 7.09–6.97 (m, 3H), 6.10 (d, J = 8.4 Hz, 1H), 5.23 (s, 2H), 3.92 (t, J = 7.2 Hz, 2H), 3.12 (t, J = 7.2 Hz, 2H), 2.47 (s, 3H), 2.24 (s, 3H); ^{13}C NMR (100 MHz, $CDCl_3$) δ 168.3, 134.9, 134.1, 133.9, 133.7, 133.6, 132.4, 132.1, 129.1, 128.8, 128.0, 127.9, 127.7, 123.10, 123.08, 118.5, 109.3, 108.4, 44.0, 38.4, 23.5, 18.5, 9.9.

K149. mp = 198°C (dec.); IR (thin film) 3447–3402 (broad), 3018, 2917, 1654, 1637, 1473 cm^{-1}; ^1H NMR (400 MHz, DMSO-d_6) δ 7.86 (br s, 3H), 7.70 (d, J = 2.0 Hz, 1H), 7.57 (s, 1H), 7.28–7.24 (m, 2H), 7.05 (dd, J = 8.4, 1.6 Hz, 1H), 6.19 (d, J = 8.4 Hz, 1H), 5.41 (s, 2H), 2.99–2.95 (m, 4H), 2.49 (s, 3H), 2.25 (s, 3H); ^{13}C NMR (100 MHz, DMSO-d_6) δ 135.5, 135.12, 135.07, 133.0, 132.5, 129.3, 128.7, 128.6, 128.3, 127.7, 122.3, 117.9, 110.5, 107.1, 44.1, 39.9, 22.7, 17.9, 10.2; HRMS (ESI$^+$) *m/z* calcd for $C_{19}H_{18}Cl_2NS$ [M-NH3]$^+$: 362.0532. Found: 362.0526. Anal. calcd for $C_{19}H_{21}Cl3N_2S$: C, 54.88; H, 5.09; N, 6.74; Found: C, 54.66; H, 4.89; N, 6.98.

REFERENCES

1. Bosch, J, Rubiralta, M, Moral, M, Ariño, J. Benzomorphan-related Compounds. Part 21. Synthesis of 7,8-Benzomorphans via 2-Aryl-4-piperidones. J. Chem. Soc. Pekin 1 1986; 1533–1539.
2. Ahlström, M. M, Ridderström, M, Zamora, I, Luthman, K. CYP2C9 Structure-Metabolism Relationships: Optimizing the Metabolic Stability of COX-2 Inhibitors. J. Med. Chem. 2007; 50:4444–4452.

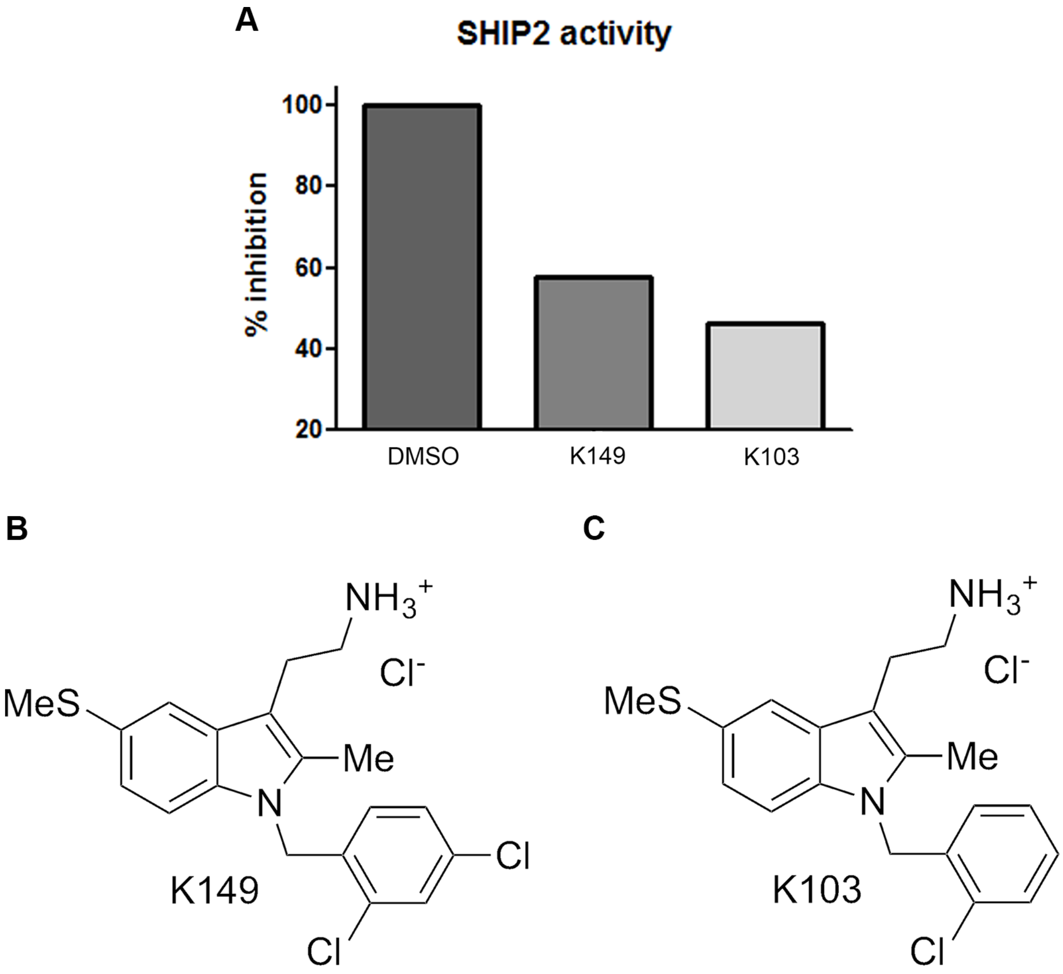

Supplementary Figure S1: Confirmation of SHIP2 activity inhibition using SHIP2 inhibitors. (**A**) Inhibition of recombinant SHIP2 by K149 as measured by Malachite Green Assay. Recombinant SHIP2 was incubated with vehicle control (DMSO) or 500 uM of the SHIP2 inhibitors K149 and K103, showing inhibition of the SHIP2 activity. (**B**, **C**) Structure formulas of the two SHIP2 inhibitors.

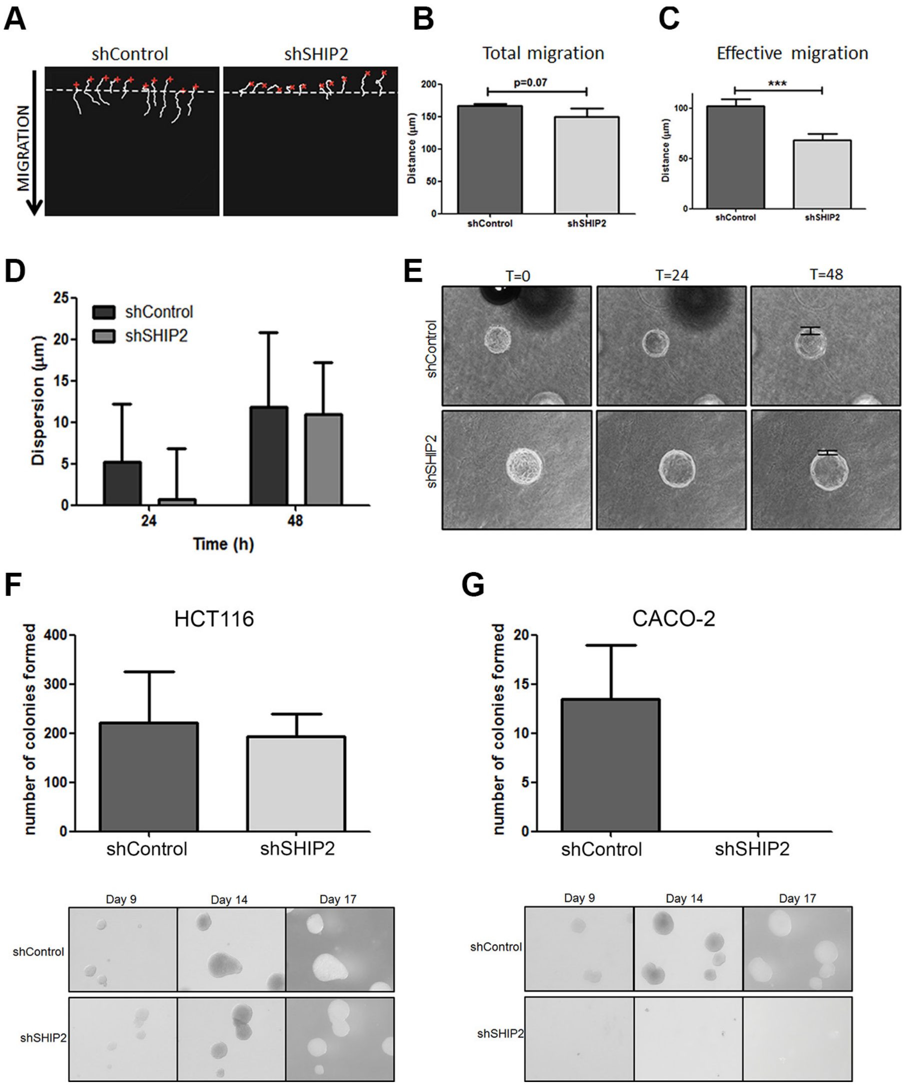

Supplementary Figure S2: SHIP2 influences cell migration and invasion. (**A**) Cell migration was measured using a ring-barrier system. CACO-2 cell migration on gelatin was tracked during 24 h, with locations being captured using time-lapse microscopy every 12 min (x = start, line = cell track). (**B**, **C**) Quantification of migrated path indicates that the total migration and effective migration were significantly reduced in SHIP2 knockdown cells. (***$P < 0.001$). (**D**) Beads were coated with either CACO-2 SHIP2 knockdown or control cells for 24 hours, and embedded in a collagen gel matrix. Cells were allowed to invade the collagen matrix, and pictures were taking at 0 h, 24 h, and 48 h (examples in **E**). The cell dispersion from the bead (arrow) into the collagen matrix (**F**, **G**) Colony formation assays of HCT116 (F) and CACO-2 (G) cells. Number of colonies were counted. Quantification is shown in upper panels, photographic examples in lower panels. was measured, and a trend towards reduced invasion was observed in SHIP2 knockdown cells. Data represents at least four beads.

Supplementary Table S1: Patient characteristics of tissue micro array stained for SHIP2

Parameter	SHIP2 Low	SHIP2 high	Overall	*P*-value
**Number of patients**	101	246	347	
**Gender (M)**	50 (50%)	122 (50%)	172 (50%)	
**Age**	64.99	66.04	65.30	
**Location**				
Left-sided	58 (62%)	168 (72%)	226 (70%)	0.112
Right-sided	35 (38%)	66 (28%)	101 (30%)	
**TNM stadium (AJCC 5)**				
Stage I	25 (25%)	40 (17%)	65 (19%)	0.294
Stage II	36 (36%)	84 (35%)	120 (36%)	
Stage III	24 (24%)	69 (29%)	93 (28%)	
Stage IV	15 (15%)	45 (19%^)	60 (18%)	
**Differentiation tumor**				
Well	20 (26%)	37 (19%)	57 (21%)	0.454
Moderate	48 (62%)	134 (69%)	182 (67%)	
Poor	9 (12%)	37 (11%)	31 (11%)	
**Dukes stadium**				
A/B	62 (62%)	126 (53%)	188 (55%)	0.290
C	23 (23%)	68 (28%)	91 (27%)	
D	15 (15%)	45 (19%)	60 (18%)	
**MSI-status**				
MSS	58 (60%)	174 (75%)	232 (71%)	**0.032**
MSI-H	13 (14%)	23 (10%)	36 (11%)	
unknown	25 (26%)	36 (15%)	61 (19%)	
**p53**				
0–25%	59 (60%)	116 (48%)	175 (51%)	**0.043**
> 25%	39 (40%)	126 (52%)	165 (39%)	
**PIK3CA**				
WT	66 (65%)	195 (79%)	261 (75%)	0.683
Mutation	10 (10%)	25 (10%)	35 (10%)	
Missing	25 (25%)	26 (11%)	51 (15%)	

Supplementary Table S2: Uni- and multivariate analysis for overall survival

	Univariate		Multivariate	
	HR	*p*-value	HR	*p*-value
**SHIP2**	1.421(1.05–1.922)	**0.023**	1.232 (0.906-1.676)	0.183
**Gender (M)**	1.304 (1.044–1.629)	**0.019**		
**Age**	1.039 (1.028–1.05)	**< 0.001**	1.049 (1.035-1.063)	**< 0.001**
**TNM stage**				
stage 1		**< 0.001**		**< 0.001**
stage 2	1.386 (0.983–1.954)		1.635 (1.069-2.500)	
stage3	2.078 (1.459–2.959)		2.279 (1.474-3.523)	
stage 4	6.142 (4.199–8.985)		6.691 (4.246-10.546)	
**Dukes' stage**				
A/B		**< 0.001**		
C	1.696 (1.3–-2.212)			
D	4.987 (3.698–6.726)			
**Differentation**	0.96 (0.825–1.117)	0.596		
**Adjuvant therapy**	1.056 (0.957–1.166)	0.279		
**Tumor location**	1.258 (0.988–1.601)	0.063		

Supplementary Video S1–S4: Downmodulation of SHIP2 results in reduced migration. Time-lapse movies (images taken every 12 min) of cell migration in CACO-2 (S1–S2) and HCT116 (S3–S4) control and knockdown cells. SHIP2 knockdown cells migrate significantly less as compared to control cells. The impairment of the directional movement is even more pronounced.
